# Supplementary material for: Venomix: a simple bioinformatic pipeline for identifying and characterizing toxin gene candidates from transcriptomic data
Source: PeerJ. 2018 Jul 31;6:e5361. doi: 10.7717/peerj.5361 (PMC6074769; doi:10.7717/peerj.5361)
Supplement: Supplemental Information 3 [file peerj-06-5361-s003.gz › FinalOutput_E-6/Venom_allergen_5_15/finaltree.pdf]

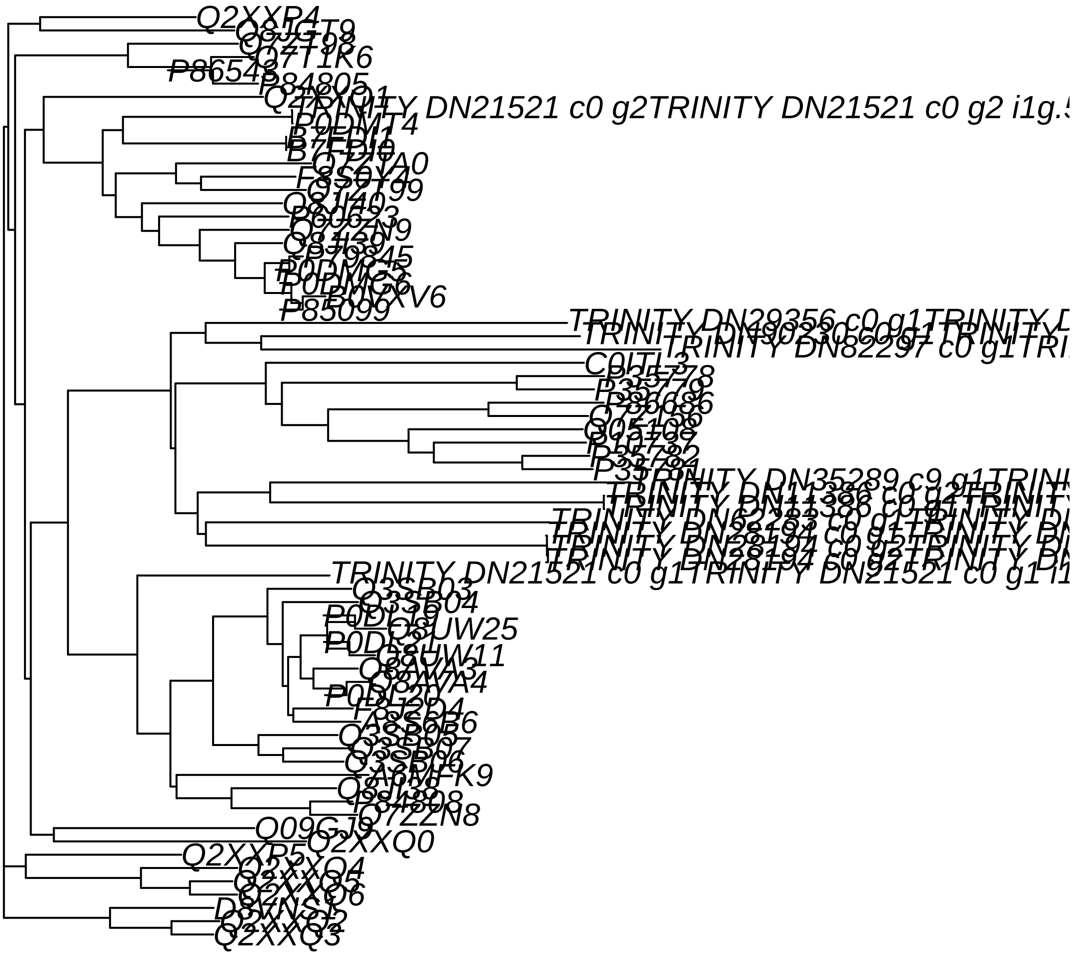
Q2XXP4
Q8JGT9
Q7ZT98
Q7T1K6
P86543
P84805
Q2XXQ1
P0DMT4
B7FDI0
Q9I9A0
F8S0Y4
Q9I9A9
Q8JJ40
P00623
Q7ZZN9
P79845
P0DMG5
P0DMG6
P0CXV6
P85099
C0ITL3
P35778
Q05108
P33782
TRINITY DN21521 c0 g1TRINITY DN21521 c0 g1 i1
Q3SB03
Q3SB04
Q8UW25
Q8UW11
Q8AVA3
Q8AVA4
P0DL20
A8S6B6
Q3SB05
Q3SB07
Q3SB06
A0A0K9
Q8JI38
P84808
Q7ZZN8
Q09GJ9
Q2XXQ0
Q2XXP5
Q2XXQ4
Q2XXQ5
Q2XXQ6
D9YNSL
Q2XXQ2
Q2XXQ3
